# Secretory Phospholipases A_2_ in Durum Wheat (*Triticum durum* Desf.): Gene Expression, Enzymatic Activity, and Relation to Drought Stress Adaptation

**DOI:** 10.3390/ijms14035146

**Published:** 2013-03-01

**Authors:** Angelo Verlotta, Maria T. Liberatore, Luigi Cattivelli, Daniela Trono

**Affiliations:** Consiglio per la Ricerca e la sperimentazione in Agricoltura, Cereal Research Centre, S.S. 16, Km 675, 71122 Foggia, Italy; E-Mails: angeloverlotta@yahoo.it (A.V.); mt1981@libero.it (M.T.L.); luigi.cattivelli@entecra.it (L.C.)

**Keywords:** drought stress, durum wheat, phospholipase A_2_

## Abstract

Phospholipases A_2_ (PLA_2_s) are known to mediate signaling cascades during plant growth and development, as well as biotic and abiotic stress responses. In this context, the present study provides extensive characterization of specific PLA_2_s in durum wheat, and assesses their involvement in durum wheat response to drought stress. In durum wheat leaves, four full-length expressed sequences encoding putative PLA_2_s were isolated and characterized as belonging to the class of secretory PLA_2_s (sPLA_2_s): *TdsPLA_2_I*, *TdsPLA_2_II*, *TdsPLA_2_III* and *TdsPLA_2_IV*. PLA_2_ activity was also detected, the characteristics of which resemble those of previously characterized plant sPLA_2_s: strong preference for phospholipids; requirement for millimolar Ca^2+^ concentrations; optimal activity at basic pH; heat stability; and inhibition by the reducing agent dithiothreitol. With drought stress imposed at both the vegetative and reproductive stages, accumulation of *TdsPLA_2_I* and *TdsPLA_2_III* transcripts, and to a lesser extent of *TdsPLA_2_IV* transcript, paralleled increased PLA_2_ activity; both transcript levels and enzymatic activity decreased as a consequence of stress recovery. Consistently, free fatty acid analysis of drought-stressed leaves revealed increased linoleate, linolenate and palmitate contents, which were reversed by plant re-watering. Overall, these findings strongly suggest that there are inducible sPLA_2_ isoforms in durum wheat that have roles in orchestrating the plant response to drought stress.

## 1. Introduction

To adapt growth and metabolism to variations in environmental conditions, plants have developed complex signaling networks to perceive environmental stimuli and then transduce the information across the plasma membrane into the cell, where it activates a specific signaling cascade. In this context, the production of lipid mediators triggered by phospholipases through the hydrolysis of membrane phospholipids has a pivotal role in plant response to environmental stress [1].

In plants, as in other organisms, there are three major classes of phospholipases that are distinguished based on their substrate cleavage site: phospholipase C (PLC), phospholipase D (PLD) and phospholipases A (PLA) [2]. While the roles of both PLC and PLD as important signaling enzymes under stress conditions have been extensively investigated [1], information about the deacylating enzymes remains very limited.

PLA_2_ (phosphatide 2-acylhydrolase, EC 3.1.1.4) is a deacylating enzyme that specifically hydrolyses glycerophospholipids at the *sn*-2 position to yield free fatty acids (FFAs) and lysophospholipids (LPLs). Both of these reaction products represent precursors for signaling molecules that are biologically active in a wide range of physiological and pathological processes. Currently, the PLA_2_ superfamily consists of several groups of enzymes that are classified into five different types on the basis of their amino acid sequence, molecular weight, Ca^2+^-dependence, and cell localization: the low-molecular-weight, secretory PLA_2_s (sPLA_2_s); the cytosolic, Ca^2+^-dependent PLA_2_s (cPLA_2_s); the cytosolic, Ca^2+^-independent PLA_2_s (iPLA_2_s); the platelet-activating factor acylhydrolases; and the lysosomal PLA_2_s [3].

In plants, to the best of our knowledge, the only specific PLA_2_s discovered belong to the type of sPLA_2_s. Less specific patatin-like PLAs have been described that show low positional specificity for the *sn-*1 and *sn*-2 C atom in their phospholipid substrates, and the ability to also hydrolyze galactolipids [4]. Even after sequencing the entire genomes of various plant species, no cPLA_2_-like genes have yet been found.

As far as the specific sPLA_2_s are concerned, these were first purified from elm seeds [5] and rice shoots [6], and more recently from castor bean leaves [7]. The cDNA for putative sPLA_2_s from rice shoots and carnation flowers were the first ones to be cloned [6,8], followed by the four sPLA_2_s of *A. thaliana*[9] and, very recently, by the five sPLA_2_s of *G. max*[10]. With progress in genome sequencing, the number of plant sPLA_2_s identified is steadily increasing. As for their animal counterparts, the plant sPLA_2_s are characterized by low molecular weights (13–18 kDa) and contain conserved regions, including twelve Cys residues that form six intramolecular disulfide bridges, a His residue in the catalytic site, a Ca^2+^-binding loop and a signal peptide for secretion [9]. According to the criteria established for animal sPLA_2_s, plant sPLA_2_s are classified as a separate group, group XI, that is divided into two clusters, XIA and XIB, on the basis of the amino acid sequence identities [3].

Plant PLA_2_s are known to be involved in a number of important physiological and pathological processes [11]. Nevertheless, in most cases, the molecular identities of the enzymes that contribute to these processes remain unknown. Thus, activities not assigned to a specific class of the PLA_2_s have been shown to have roles in plant responses to auxin, wounding, pathogen attack and elicitors [11], programmed cell death [12] and cold acclimation [13]. In other cases, this information relates to non-specific lipid acyl hydrolases, such as the patatin-like PLAs, that have been shown to have roles in cellulose deposition and cell elongation [14–16], as well as in signaling cascades triggered by wounding, pathogens and elicitors, and in abiotic stress responses [4].

With regard to specific sPLA_2_s, numerous lines of evidence have demonstrated roles for this class of enzymes in flowering [8], auxin-mediated cell elongation and shoot gravitropism [17], light signal transduction in guard cells [18], PIN protein trafficking [19], pollen development and germination [20]. Only recently has the first evidence been reported about the involvement of this class of PLA_2_s in plant responses to both biotic and abiotic stresses. sPLA_2_s have been found to be involved in plant response to both bacterial [21] and yeast [22] infections. Moreover, Ryu and co-workers demonstrated that over-expression of the *AtsPLA_2_α* and *AtsPLA_2_β* genes in transgenic plants of *A. thaliana* and *N. tabacum* increases their resistance not only to a variety of pathogen infections, but also to salt stress [23]. In line with these findings, Singh and co-workers reported the up-regulation of the *OssPLA_2_α* gene in rice seedlings exposed to drought stress [24].

Among the environmental stresses, water deficit represents one of the major factors that limit plant growth and productivity. Durum wheat cultivation faces drought conditions very often in various arid and semi-arid regions, such as the Mediterranean regions that produce some 75% of the world’s durum grain [25]. Thus, in order to sustain the yields and yield stability of this important crop species, adaptation to drought stress represents an important breeding target. In this context, there is the need for the identification of the mechanisms involved in durum wheat responses to water deficit. Therefore, the aim of the present study was to investigate the existence, in durum wheat, of specific sPLA_2_s and to establish their roles in durum wheat adaptation to drought stress.

## 2. Results

### 2.1. Isolation and Characterization of Durum Wheat Full-Length sPLA_2_ cDNAs and Gene Expression Analysis in Different Tissues

The full-length *sPLA_2_* transcripts were isolated after amplification of cDNA from the youngest fully expanded leaves of durum wheat plants at the tillering stage. Four expressed sequences were isolated that ranged between 420 and 513 bp (from start to stop codon), and they were designated as *TdsPLA_2_I*, *TdsPLA_2_II*, *TdsPLA_2_III* and *TdsPLA_2_IV* (GenBank: JX021445, JX021446, JX021447 and JX021448, respectively) on the basis of their similarities with the coding sequences deduced from the Os02g0831700 (*OssPLA_2_I*), Os03g0261100 (*OssPLA_2_II*), Os03g0708000 (*OssPLA_2_III*) and Os11g0546600 (*OssPLA_2_IV*) genes.

**Figure 1:**
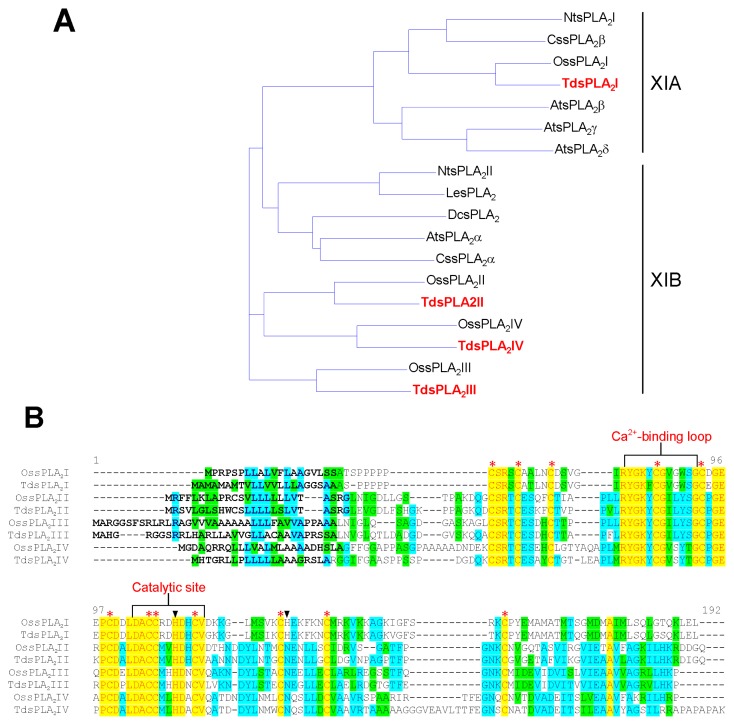

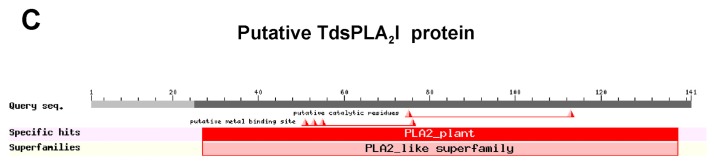
Analysis of the deduced amino acid sequences of sPLA_2_s from durum wheat leaves. (**A**) Phylogenetic tree of the deduced amino acid sequences of the four TdsPLA_2_s and some other plant sPLA_2_s. The GenBank accession numbers are as follows: *A. thaliana* isoform α (AtsPLA_2_α), β (AtsPLA_2_β), γ (AtsPLA_2_γ) and δ (AtsPLA_2_δ), At2g06925, At2g19690, At4g29460 and At4g29470, respectively; carnation (DcsPLA_2_), AF064732; durum wheat isoform I (TdsPLA_2_I), II (TdsPLA_2_II), III (TdsPLA_2_III) and IV (TdsPLA_2_IV), JX021445, JX021446, JX021447 and JX021448, respectively; orange isoform α (CssPLA_2_α) and β (CssPLA_2_β), GU075396 and GU075398, respectively; rice isoform I (OssPLA_2_I), II (OssPLA_2_II), III (OssPLA_2_III) and IV (OssPLA_2_IV), Os02g0831700, Os03g0261100, Os03g0708000 and Os11g0546600, respectively; tobacco isoform I (NtsPLA_2_I) and II (NtsPLA_2_II), AB190177 and AB190178, respectively; tomato (LesPLA_2_), AI487873. (**B**) Alignments between the deduced amino acid sequences of rice and durum wheat sPLA_2_s. The conserved domains with homology to the Ca^2+^-binding loop and the active site motifs of other known plant sPLA_2_s are indicated. Each of the twelve conserved Cys residues is marked with an asterisk. Triangles indicate the catalytic dyad. The signal peptides are highlighted in bold. The program used to produce phylogenetic tree and sequence alignment was the Vector NTI Suite software (version 9.0; Life Technology, Carlsbad, CA, USA). (**C**) Typical domains in TdsPLA_2_I as identified by the NCBI Conserved Domain Search Database [27]. The same result was obtained by using as query the amino acid sequences deduced from the other three TdsPLA_2_ isoforms.

Alignment of the deduced amino acid sequences of the four TdsPLA_2_s with some other known plant sPLA_2_s (Figure 1A) revealed that, analogous to the corresponding isoforms in rice [26], TdsPLA_2_I can be assigned to the cluster XIA, whereas the three other isoforms belong to the cluster XIB. As shown in Figure 1B, TdsPLA_2_I, TdsPLA_2_II, TdsPLA_2_III and TdsPLA_2_IV sequence lengths were 141, 157, 162 and 170 amino acids, respectively, with the molecular masses ranging from 15 to 17 kDa. All of the deduced proteins have typical features that have already been identified in other sPLA_2_s from both animal and plant sources (Figure 1B,C) [9]: (i) the signal peptide for secretion in the N-terminal region; (ii) the PLA_2_ signature domain that includes the Ca^2+^-binding loop and the catalytic site; and (iii) twelve Cys residues that have the potential to form six intramolecular disulfide bridges. Sequence alignment also revealed that, paralleling the OssPLA_2_ isoforms [26], the Asp residue of the highly conserved His/Asp catalytic dyad of the animal counterpart is replaced by a His residue in the durum wheat sPLA_2_ isoform I, and by an Asn residue in all of the other durum wheat sPLA_2_ isoforms. The intra-*TdsPLA_2_* sequence comparisons yielded between 48.0% and 64.8% identity at the transcript level and between 24.7% and 49.7% identity at the protein level, with the TdsPLA_2_I isoform showing the lowest identity with all of the other isoforms at both transcript and amino acid level. This is expected, as this is the only durum wheat sPLA_2_ isoform that belongs to the cluster XIA. In spite of the low identity shared by these overall amino acid sequences, the conserved regions corresponding to the Ca^2+^-binding loop and the catalytic site were found to share 54.5% and 72.7% identities, respectively, among the four durum wheat sPLA_2_ isoforms.

**Figure 2:**
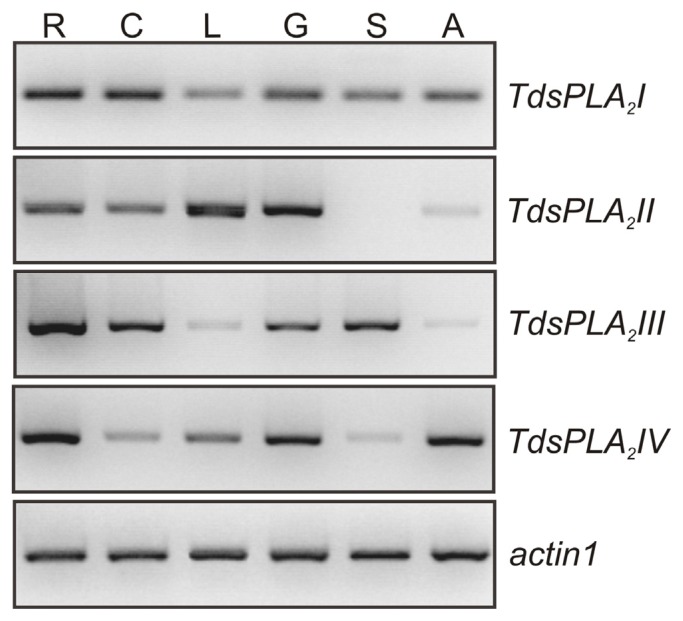
Tissue-specific expression of the *TdsPLA_2_* genes. Representative expression analysis of *TdsPLA_2_I*, *TdsPLA_2_II*, *TdsPLA_2_III*, *TdsPLA_2_IV* and *actin1* genes carried out by using the specific primer pairs reported in Table 1. The figures of gel separation are presented in inverted colors. R, root; C, culm; L, leaf; G, glume; S, seed; A, awn.

As shown in Figure 2, the four *TdsPLA_2_* genes were expressed in durum wheat tissues, although at different levels and with different tissue specificities. *TdsPLA_2_I* was expressed in all of the plant organs analyzed, with the highest levels in root and culm. *TdsPLA_2_II* was expressed in root and culm, and at very high levels in leaf and glume; in contrast, the expression levels of the *TdsPLA_2_II* gene were very low, and even absent, in awn and seed. Transcripts corresponding to *TdsPLA_2_III* were more abundant in root, but were also present in culm, glume and seed, and to a lesser extent, in leaf and awn. *TdsPLA_2_IV* was expressed at high levels in roots, glume and awn, whereas there were low *TdsPLA_2_IV* transcript levels detected in culm and seed.

### 2.2. Identification and Biochemical Characterization of a Ca^2+^-Dependent PLA_2_ Activity in Durum Wheat Leaves

In the light of the results obtained at the molecular level, an investigation was carried out to evaluate the existence, in durum wheat leaves, of a PLA_2_ activity resembling the characteristics typical of known sPLA_2_s. The enzymatic assays were carried out on crude extracts from the fully expanded leaves of durum wheat plants at the tillering stage.

**Figure 3:**
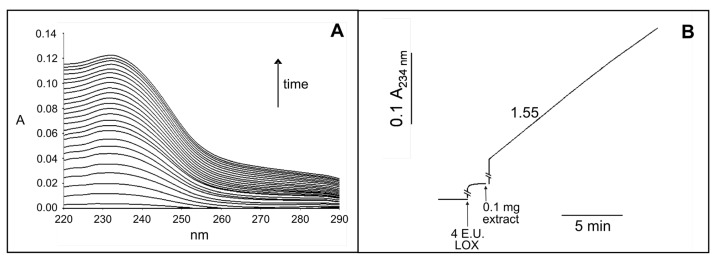
Assay of PLA_2_ activity in the crude extract from durum wheat leaves. (**A**) PLA_2_ activity monitored as appearance of the typical hydroperoxide spectrum. The reaction mixture contained 2 mM CaCl_2_, 1.5 mM PC_LIN_ and 4 E.U. LOX in 2 mL 50 mM Na borate buffer, pH 9.0; the reaction was started by the addition of 0.1 mg of crude leaf extract. The absorption spectra were recorded every 20 s; (**B**) PLA_2_ activity monitored as time course at 234 nm. The reaction mixture contained 2 mM CaCl_2_ and 1.5 mM PC_LIN_ in 2 mL 50 mM Na borate buffer pH 9.0; 4 E.U. LOX and 0.1 mg of crude leaf extract were added at the time indicated. The number on the trace refers to E.U. per gram of dry weight.

As shown in Figure 3A, the addition of crude leaf extract (0.1 mg) to a reaction mixture containing 2 mM CaCl_2_, 4 enzymatic units (E.U.) lipoxygenase (LOX) and 1.5 mM 1-palmitoyl-2-linoleoyl-*sn-*glycero- 3-phosphocholine (PC_LIN_) induced an absorbance increase in the UV region with a maximum of 234 nm. This was due to the generation of free linoleate and its conversion into the corresponding hydroperoxide containing a conjugated diene. Further investigation was carried out by measuring continuously at 234 nm the hydroperoxidation of the linoleate released from the PC_LIN_. As shown in Figure 3B, an absorbance increase was observed as a consequence of the sequential additions of 4 E.U. LOX and 0.1 mg crude leaf extract to a reaction mixture containing 2 mM CaCl_2_ and 1.5 mM PC_LIN_. A PLA_2_ activity equivalent to 1.55 E.U. g^−1^ dry weight was determined.

**Figure 4:**
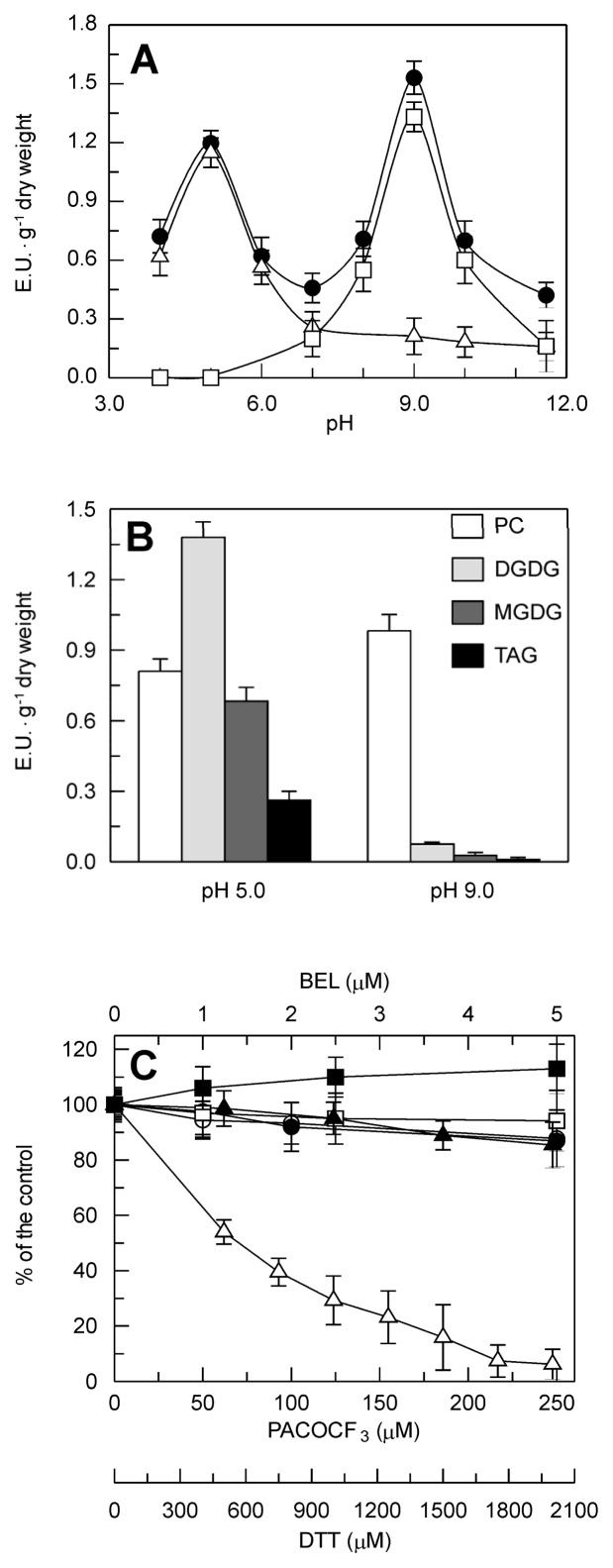
Biochemical characterization of the PLA_2_ activity detected in the crude extract from durum wheat leaves. (**A**) Effect of pH, Ca^2+^ and heat inactivation. Measurements were carried out at 25 °C in the presence of 2 mM CaCl_2_ (●), 10 mM EGTA (Δ) or denatured (15 min at 100 °C) crude leaf extract (□). (**B**) Substrate preference. PC, phosphatidylcholine; DGDG, digalactosyldiacylglycerol; MGDG, monogalactosyldiacylglycerol; TAG, triacylglycerol. (**C**) Sensitivity to inhibitors. Open symbols refer to the PLA_2_ activity detected at pH 9.0, closed symbols refer to the PLA_2_ activity detected at pH 5.0. (■,□) BEL, bromoenol lactone, iPLA_2_ inhibitor; (●, ○) PACOCF_3_, palmityl trifluoromethyl ketone, cPLA_2_ and iPLA_2_ inhibitor; (▲, Δ) DTT, dithiothreitol, sPLA_2_ inhibitor. Data are means ± SD (*n* = 3).

A set of experiments was carried out to make a first biochemical characterization of the PLA_2_ activity. For the pH profile (Figure 4A), the activity of the crude extract showed two peaks, the smaller at pH 5.0, and the larger at pH 9.0. The two peaks showed different behaviors with respect to Ca^2+^ requirements and heat inactivation. Indeed, the addition to the reaction mixture of 10 mM ethylene glycol-bis(2-aminoethylether)-*N,N,N′,N′*-tetraacetic acid (EGTA), a Ca^2+^ chelator, resulted in the disappearance of the peak at pH 9.0, whereas the peak at pH 5.0 was not affected. In contrast, the denaturation of the crude leaf extract (15 min at 100 °C) completely abolished the peak at pH 5.0, whereas that at pH 9.0 was only slightly affected.

To provide further insight into the nature of the activities detected at both of these pH optima, an investigation was carried out to assess: (i) the preference for different lipid substrates, and (ii) the sensitivity to specific PLA_2_ inhibitors. As shown in Figure 4B, all the four lipids tested were substrates of the crude leaf extract activity at pH 5.0, with the highest rate for digalactosyldiacylglycerol (DGDG), followed by phosphatidylcholine (PC), monogalactosyldiacylglycerol (MGDG) and triacylglycerol (TAG). The activity at pH 5.0 was insensitive to Ca^2+^, whatever the substrate used (data not shown). At pH 9.0, the highest activity was seen with PC, whereas very low rates were observed with all of the other substrates, probably ascribable to the residual activity, at this pH value, of the enzyme/s responsible for the peak at pH 5.0; consistently, while the activity detected at pH 9.0 using PC as substrate was almost completely abolished by 10 mM EGTA (see Figure 4A), the activity toward all of these other substrates was not affected by the presence of the Ca^2+^ chelator (data not shown).

The effects of the following specific PLA_2_ inhibitors were also tested on the activities detected at pH 5.0 and pH 9.0 (Figure 4C): the disulfide bond-reducing agent dithiothreitol (DTT), an inhibitor of animal sPLA_2_s; palmityl trifluoromethyl ketone (PACOCF_3_), an inhibitor of animal cPLA_2_s and iPLA_2_s; bromoenol lactone (BEL), an inhibitor of animal iPLA_2_s [28]. The crude leaf extract activity measured at pH 9.0 was unaffected by increasing concentrations of PACOCF_3_ and BEL; in contrast, at pH 9.0, the crude leaf extract activity was inhibited in a dose-dependent manner by DTT, which decreased to almost zero at 2 mM DTT. For the activity detected in durum wheat leaves at acidic pH range, none of these PLA_2_s inhibitors had any effects.

Altogether, these findings suggest that the activity detected in durum wheat leaves at basic pH values could be ascribable to the presence in this tissue of one or more specific sPLA_2_ isoforms, as the properties of this activity resemble those of other known plant sPLA_2_s: preference for phospholipid substrate, optimum at basic pH, Ca^2+^-dependence, heat stability, and inhibition by the disulfide bond-reducing agent DTT. In contrast, the activity that peaked at the acidic pH of 5.0 is probably due to one or more generic acyl hydrolases. Consequently, to better characterize the specific Ca^2+^-dependent PLA_2_ activity detected at basic pH, which is hereafter referred to as the durum-wheat-leaves-PLA_2_ (DWL-PLA_2_), all of the experiments reported below were carried out at pH 9.0 in the presence of 2 mM CaCl_2_; moreover, to give a measure exclusive of the Ca^2+^-dependent PLA_2_ activity, the residual activity measured in the presence of 10 mM EGTA was consistently subtracted.

**Figure 5:**
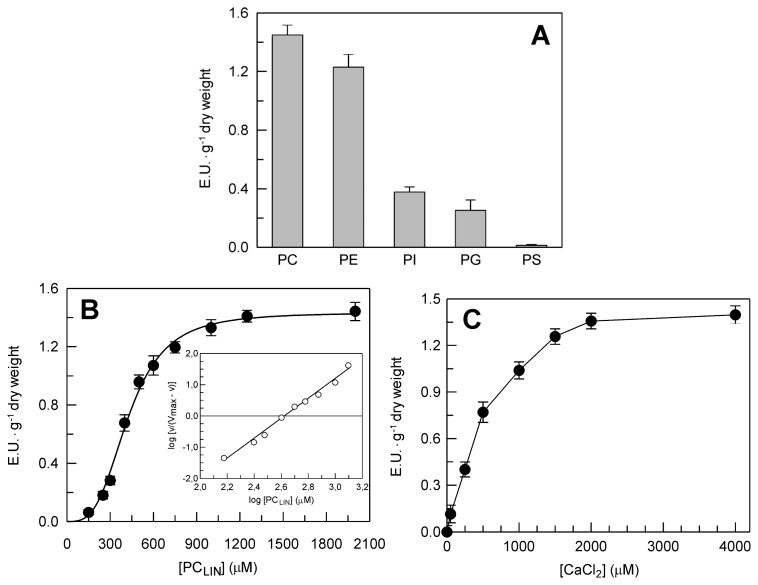
Substrate and Ca^2+^ dependence of DWL-PLA_2_ detected at pH 9.0 in the crude extract from durum wheat leaves. (**A**) Headgroup selectivity. PC, phosphatidylcholine; PE, phosphatidylethanolamine; PI, phosphatidylinositol; PG, phosphatidylglycerol; PS, phosphatidylserine; (**B**) Dependence on substrate concentration. The assays were carried out at different PC_LIN_ concentrations ranging between 150 and 2000 μM; (**C**) Dependence on Ca^2+^ concentration. The assays were carried out at different CaCl_2_ concentrations ranging between 50 and 2000 μM. The point at zero Ca^2+^ concentration was obtained in the presence of 10 mM EGTA. Data are means ± SD (*n* = 3).

The DWL-PLA_2_ activity showed a marked preference for PC and phosphatidylethanolamine (PE), as compared to phosphatidylinositol (PI), phosphatidylglycerol (PG) and phosphatidylserine (PS) (Figure 5A). In light of this, PC_LIN_ was used to evaluate the dependence of DWL-PLA_2_ on substrate concentration. As shown in Figure 5B, the reaction rate showed a sigmoidal dependence on substrate concentration. The K_0.5_ (substrate concentration which gives half maximal rate with sigmoidal kinetics) and the V_max_ were 430 ± 37 μM (SD) and 1.43 ± 0.038 E.U. g^−1^ dry weight, respectively. The Hill plot (Figure 5B, inset) gave a coefficient of 3.29. The sigmoidal dependence of the DWL-PLA_2_ rate might depend on the presence in the crude leaf extract of more than one PLA_2_ isoform, each of which would have different K_m_ values. Furthermore, it should be noted that interfacial effects between substrate and enzyme are important in PLA_2_ catalysis [29], and this might also, at least in part, explain these unusual kinetics.

The DWL-PLA_2_ activity continuously increased with increasing Ca^2+^ concentrations, with a plateau at 2 to 4 mM Ca^2+^, even though 300 μM Ca^2+^ was sufficient to reach 50% of the maximal activity (Figure 5C).

### 2.3. Evaluation of TdsPLA_2_ Gene Expression and DWL-PLA_2_ Activity in Durum Wheat Leaves at Different Developmental Stages

**Figure 6:**
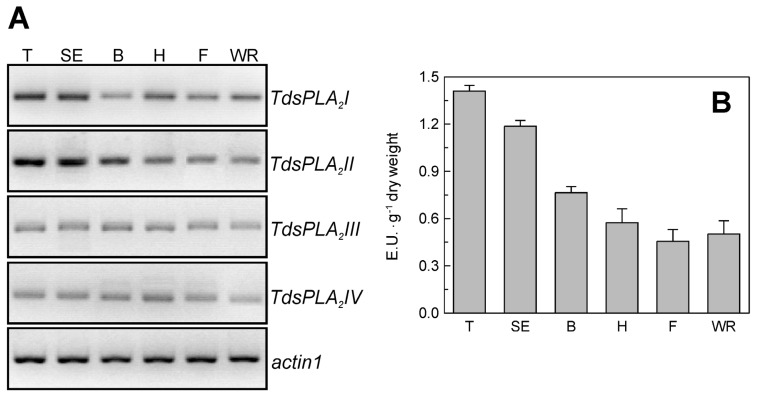
Effect of growth stage on *TdsPLA_2_* gene expression and DWL-PLA_2_ activity in durum wheat leaves. (**A**) Representative expression analysis of *TdsPLA_2_I*, *TdsPLA_2_II*, *TdsPLA_2_III*, *TdsPLA_2_IV* and *actin 1* genes carried out by using the specific primer pairs reported in Table 1. The figures of gel separation are presented in inverted colors; (**B**) DWL-PLA_2_ activity. Data are means ± SD (*n* = 3). T: tillering, SE: stem elongation. B, booting; H, heading; F, flowering; WR, kernel watery ripening.

A set of experiments was carried out to evaluate the transcript profile of the four *TdsPLA_2_* genes and the levels of the DWL-PLA_2_ activity in the youngest fully expanded leaf of durum wheat plants at different stages of development. The results obtained are reported in Figure 6. All four of the *TdsPLA_2_* genes decreased in their steady state transcript levels along with plant development (Figure 6A). In line with the overall expression pattern, DWL-PLA_2_ activity decreased with increasing plant age, with the exception of a small increase at the watery ripening stage (Figure 6B).

### 2.4. Evaluation of the Effect of the Drought Stress on TdsPLA_2_ Gene Expression and DWL-PLA_2_ Activity in Durum Wheat Leaves

**Figure 7:**
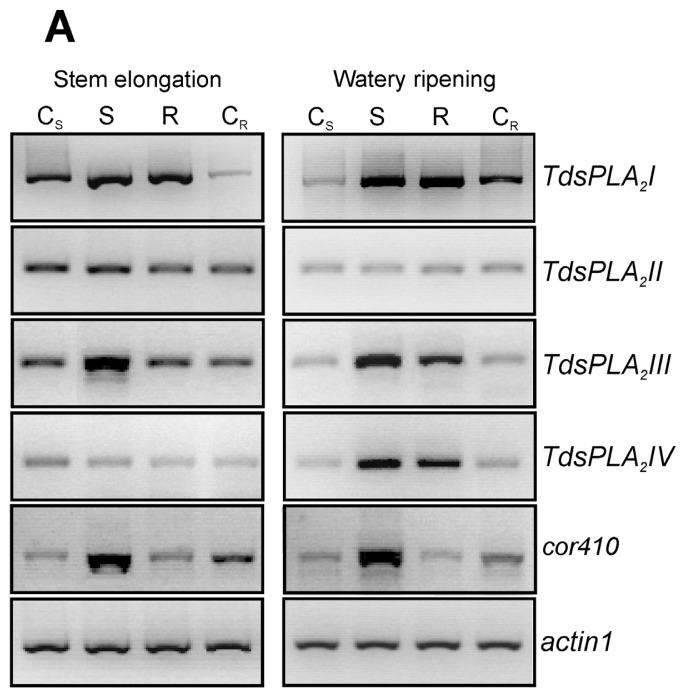

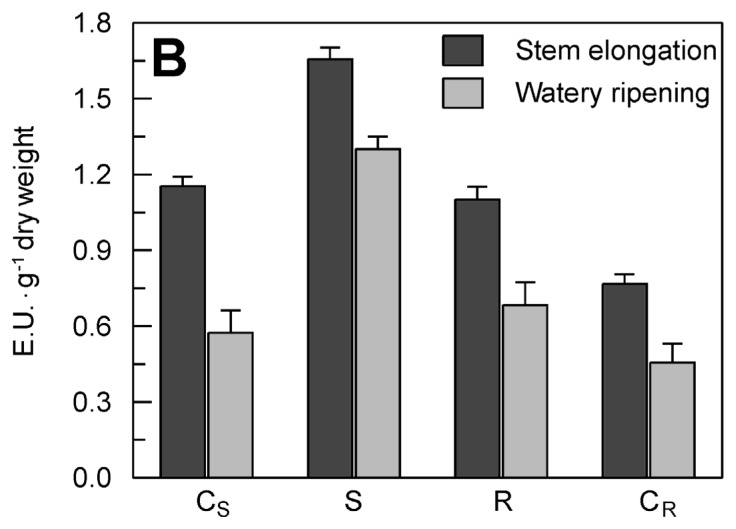
Effect of drought stress on *TdsPLA_2_* gene expression and DWL-PLA_2_ activity in durum wheat leaves. (**A**) Representative expression analysis of *TdsPLA_2_I*, *TdsPLA_2_II*, *TdsPLA_2_III*, *TdsPLA_2_IV*, *cor410* and *actin 1* genes carried out by using the specific primer pairs reported in Table 1. The figures of gel separation are presented in inverted colors; (**B**) DWL-PLA_2_ activity. Data are means ± SD (*n* = 3). C_S_: control of the stress, S: stress, R: recovery, C_R_: control of the recovery.

The effect of dehydration imposed at the vegetative (stem elongation) and reproductive (kernel watery ripening) stages was evaluated on both the transcript levels of the four *TdsPLA_2_* genes and the DWL-PLA_2_ activity in the fully expanded leaves (Figure 7).

The up-regulation of the dehydrin gene *cor410* confirmed the correct imposition of the drought stress (Figure 7A). Among the four *TdsPLA_2_* genes, both *TdsPLA_2_I* and *TdsPLA_2_III* were up-regulated under drought stress at both developmental stages. Induction was also observed in the expression of the *TdsPLA_2_IV* gene when the drought stress was imposed at the kernel watery ripening stage, while no variations were seen in the response to dehydration at transcript level for the *TdsPLA_2_II* gene. Stress recovery resulted in decreased transcript levels of the *TdsPLA_2_III* gene, which was more evident at stem elongation, when it reached values comparable to those of the control, than at kernel watery ripening, when it remained slightly higher compared to the corresponding control. Rehydration of the plants at kernel watery ripening also induced a slight decrease in the transcript levels of the *TdsPLA_2_IV* gene. In contrast, the stress recovery at both of these developmental stages did not affect the transcript levels of the *TdsPLA_2_I* gene, which remained comparable to those observed under stress.

In line with the expression profile, a strong increase was also observed in DWL-PLA_2_ activity as a consequence of the water stress imposition at both developmental stages (Figure 7B): by about 44% at stem elongation, and by 140% at kernel watery ripening. Stress recovery led to a decrease in the DWL-PLA_2_ activity, which, however, remained higher than that measured in the corresponding controls at both developmental stages.

As far as the PLA_2_ activity detected at pH 5.0, it was found to be not affected by stress imposition at both developmental stages (data not shown).

### 2.5. Evaluation of the Effect of the Drought Stress on FFA Content in Durum Wheat Leaves

**Figure 8:**
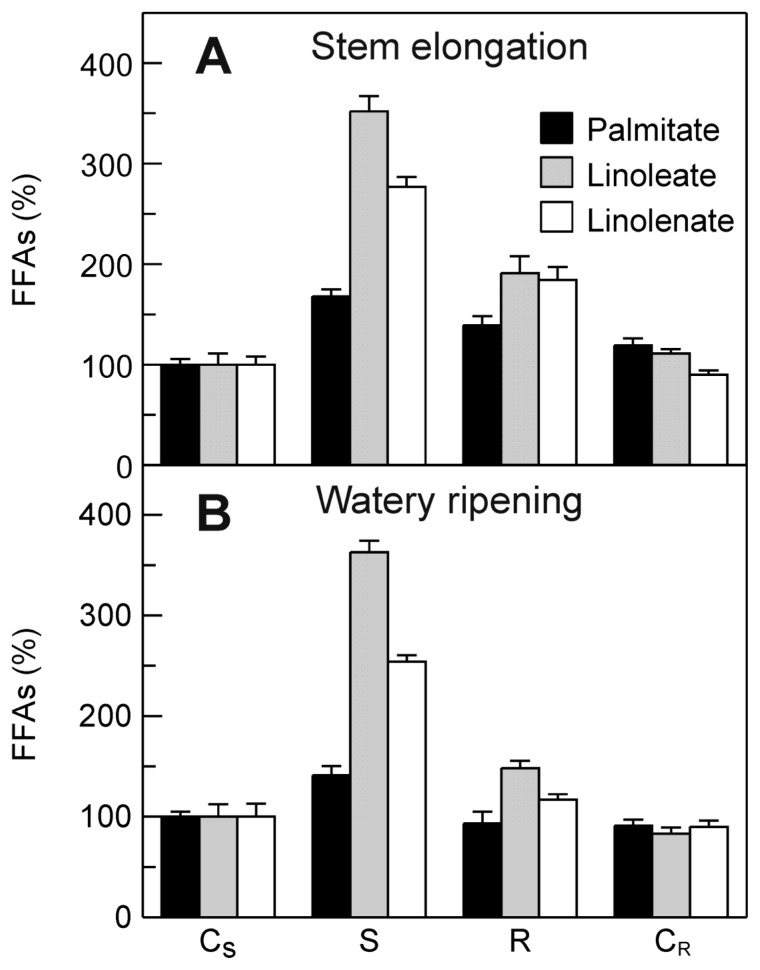
Effect of drought stress on FFA content in durum wheat leaves. Measurements were carried out at stem elongation (**A**) and watery ripening (**B**). C_S_: control of the stress; S: stress; R: recovery; C_R_: control of the recovery. Relative amounts of each FFA are expressed as percentage of the amount obtained for the C_S_ taken as 100%. Data are means ± SD (*n* = 3).

In the light of the stress-induced increases in both the *TdsPLA_2_* transcript levels and the DWL-PLA_2_ activity, an investigation was carried out to assess the effect of dehydration on the FFA content of the durum wheat leaves. As shown in Figure 8, the relative amounts of the FFAs were increased as a consequence of the stress imposition. Under stress, the levels of free linoleate and linolenate showed greater increases, of about 3.5-fold and 2.8-fold, respectively, at stem elongation, and about 3.6-fold and 2.5-fold, respectively, at watery ripening. A less evident stress-induced increase was observed for free palmitate: about 1.8-fold and 1.4-fold at stem elongation and watery ripening, respectively. Stress recovery caused a strong decrease in the FFA levels, although they remained higher when compared with the corresponding control, in particular at stem elongation.

## 3. Discussion

### 3.1. Identification and Characterization of the sPLA_2_ Genes and the DWL-PLA_2_ Activity in Durum Wheat

On the basis of the molecular analysis, we propose that a gene family exists in durum wheat encoding four putative sPLA_2_ isoforms. This statement is also supported by a BLAST search at the Wheat Sequence Repository [30] where three genomic contigs for each *sPLA_2_* cDNA sequence, one on each genome of the hexaploid wheat, have been identified. *TdsPLA_2_I* matched genomic sequences on chromosomes 2AL, 2BL and 2DL; *TdsPLA_2_II* on chromosomes 4AS, 4BL and 4DL; *TdsPLA_2_III* on chromosomes 4AL, 4BS and 4DS, and *TdsPLA_2_IV* on chromosomes 7AL, 7BL and 7DL. Two copies for each *TdsPLA_2_* gene should, therefore, be present in the durum wheat genome, and our results show that one copy is expressed, while the other one either is not expressed or its transcript is undistinguishable to the one cloned due to the high level of sequence identity shared by the two copies in their coding regions. As a consequence, the expression levels observed for each TdsPLA_2_ isoform are the result of the transcript abundance of both the two copies, as the primer pairs employed do not discriminate between them; regardless, estimation of the relative contribution of each copy is not relevant for the purpose of the present study, since the two copies should give rise to putative protein products with almost identical sequence and structure and, consequently, with similar functional features.

Similar to other known plant sPLA_2_s [26], TdsPLA_2_s can be sorted into the two clusters, XIA and XIB, contain twelve conserved Cys residues and a signal peptide at the N-terminus. On the basis of an *in silico*-based analysis, TdsPLA_2_I, TdsPLA_2_II and TdsPLA_2_IV are predicted to be secreted into the extracellular space. Transient expression in onion epidermal cells revealed that AtsPLA_2_β and AtsPLA_2_γ are indeed secreted into the extracellular space [9]. As far as TdsPLA_2_III, the bioinformatic tools predict TdsPLA_2_III to be targeted to mitochondria with a probability ranging between 0.4 and 0.9. At this regard, a great deal of evidence has been reported about the existence in mammalian mitochondria of both secretory and Ca^2+^-independent PLA_2_s [31]; as far as plant mitochondria, only recently has an acyl hydrolase activity resembling the characteristics of a PLA_2_ activity been detected in mitochondria purified from durum wheat seedlings [32]. Analysis of the transcript levels revealed that, with the exception of the *TdsPLA_2_II* transcript in seeds, the four *TdsPLA_2_* genes are expressed in all of the tissues of durum wheat plants examined. Similar results have been reported for the *AtsPLA_2_α*, *AtsPLA_2_β* and *AtsPLA_2_γ* genes, and the *Nt1PLA_2_* and *Nt2PLA_2_* genes, the transcripts of which have been detected in several tissues [9,33]. This is expected, considering the involvement of this class of enzyme in a variety of cellular processes in plants [9].

The PLA_2_ enzymatic activity detected in durum wheat leaves at pH 9.0, which is referred to as the DWL-PLA_2_ activity, presents biochemical characteristics that resemble those of previously characterized sPLA_2_s from plants and animals. Firstly, the enzyme(s) responsible for this DWM-PLA_2_ activity function as PLA_2_s, rather than as non-specific acyl hydrolases, as the hydrolytic activity is exerted exclusively against phospholipids, instead of galactolipids and triglycerides, with strong preference for PC and PE compared to PI, PG and PS. In contrast to animal sPLA_2_s that are selective for anionic phospholipids, most of the plant sPLA_2_s prefer zwitterionic phospholipids, such as PC and PE [9,34,35]. It has been assumed that the substrate preference of plant sPLA_2_s represents an adaptation to the difference in the natural phospholipid compositions between animal and plant membranes [26], with the latter containing mainly PC and PE [36]. Moreover, in addition to the use of a phospholipid as substrate, sPLA_2_s require Ca^2+^ as a cofactor for catalysis. Similarly, DWL-PLA_2_ activity was completely suppressed in the presence of EGTA and required millimolar Ca^2+^ concentrations to reach maximal activity, thus resembling the behavior of the animal counterpart [3] and of most of the sPLA_2_s identified in plants [5,8,9,34,35].

Two other typical features of known plant sPLA_2_s are also shown by DWL-PLA_2_: (i) the optimum at alkaline pH, which has already been reported for purified [5] and recombinant [9,35,37] sPLA_2_s from different plant sources; and (ii) the structural stability, as demonstrated by the resistance of DWL-PLA_2_ activity to high temperatures (87% of the activity detected at pH 9.0 was retained after treatment of the crude leaf extract at 100 °C for 15 min). AtsPLA_2_α and AtsPLA_2_β retained 80% to 95% of their enzyme activities following 5 min treatment in boiling H_2_O [9], and a similar result was obtained for sPLA_2_ purified from seeds of elm [5]. Previous findings with animal sPLA_2_s indicate that the structural stability of this class of PLA_2_s might be ascribable to the high number of disulfide bonds [9]. Consistent with this, our results clearly show that the DWL-PLA_2_ activity is inhibited in a dose-dependent manner by DTT, thus suggesting that one or more intramolecular disulfide bridges will be present in the enzyme(s) responsible for DWL-PLA_2_ activity. Overall, the biochemical characterization of the DWL-PLA_2_ activity strongly suggests that it is ascribable to one or more isoforms encoded by the *TdsPLA_2_* genes. This hypothesis is strengthened by the observation that plant senescence, as well as drought stress, causes fluctuations in the enzyme activity that are comparable to those in the overall expression levels of these *TdsPLA_2_* genes.

In durum wheat leaves, we also detected a generic acyl hydrolase activity that shows its optimum at acidic pH. A class of acyl hydrolases typical of the plant kingdom is seen in the patatin-related PLAs [4]. Similar to the activity detected in durum wheat leaves, patatin-related PLAs purified from other plant sources show Ca^2+^-independent and heat-sensitive activity. However, in contrast to the activity detected in durum wheat leaves, this class of enzymes is active on phospholipids and galactolipids and inactive towards diacylglycerol and triacylglycerol [11]; moreover, it displays a pH optimum at 7–8 and is strongly inhibited by several known PLA_2_ inhibitors [14,38,39]. Therefore, the possibility that the activity detected in durum wheat leaves at acidic pH is a generic acyl hydrolase different from the patatin-related PLAs cannot be excluded. This question merits further investigation in view of the important role played by different members of the acyl hydrolase family in membrane degradation and signaling in plants [11].

### 3.2. Effect of Drought Stress on TdsPLA_2_ Gene Expression, DWL-PLA_2_ Activity and FFA Release

Previous studies carried out on durum wheat have shown that water shortage during both the vegetative and reproductive stages can strongly affect growth and productivity of this crop species [40,41]. In light of this, stem elongation and kernel watery ripening were chosen as the vegetative and reproductive stages, respectively, at which to evaluate the involvement of sPLA_2_s in durum wheat response to water deficit, both at the molecular and biochemical levels.

Our results show that, in durum wheat leaves, DWM-PLA_2_ activity is modulated by water supply; contrarily, no effect was detected on the activity of the generic acyl hydrolases responsible for the activity detected at pH 5.0. The variations of the DWL-PLA_2_ activity observed under drought stress and recovery followed the overall expression pattern of the *TdsPLA_2_* genes. Such a closely coupled relationship strongly suggests that the up-regulation of the *TdsPLA_2_I*, *TdsPLA_2_III* and *TdsPLA_2_IV* genes and the consequent increase in the *de novo* synthesis of the corresponding sPLA_2_ isoforms represents the molecular mechanism behind this increase in DWL-PLA_2_ activity under stress. However, in addition to the regulation of gene expression, post-translational modifications that can affect the functionality of the TdsPLA_2_ isoforms cannot also be excluded. One possible mechanism might be the activation of the sPLA_2_ enzyme(s) due to activator factors, such as Ca^2+^ and pH, which are known to have key roles in plant defense responses against stress [42]. The resting Ca^2+^ concentration in the extracellular environment and in the intracellular stores ranges between 1 and 10 mM, whereas in the cytoplasm it is maintained in the nanomolar range [43]; thus, it is reasonable to assume that the TdsPLA_2_ isoforms responsible for DWL-PLA_2_ might be only partially activated depending on the specific free Ca^2+^ levels, and that transient elevation of Ca^2+^ levels due to stress signals [43] could represent a way to regulate them. The same goes for the pH. TdsPLA_2_ isoforms responsible for DWL-PLA_2_ require, to reach the maximal activity, pH values higher than those normally present in the extra- and intra-cellular compartments (around 5.5 and 7.5, respectively, [42]); it is thus plausible that they may be activated by alkalinization, that is known to occur under biotic and abiotic stresses in different compartments from several plant species [42,44] including cereals [45].

In terms of the physiological meaning of these data, they are consistent with the information available in the literature concerning the involvement of lipid acyl hydrolases in plant response to environmental stresses, in general, and to water stress, in particular. With respect to the latter, several evidences have been reported about the role of patatin-related PLAs in plant response to drought [46–48]. Our results show that, in durum wheat, specific sPLA_2_s rather than generic acyl hydrolases play the major role in response to drought stress. In line with this observation, evidence exist in the literature that sPLA_2_ genes, *CssPLA_2_α* and *CssPLA_2_β*, are involved in post-harvest peel pitting, a water stress-related disorder in citrus fruit [49]. Interestingly, these two genes are orthologous with the *TdsPLA_2_III* and *TdsPLA_2_I* genes, respectively, that are up-regulated in durum wheat plants under water deficit. Similar to our findings, these authors reported that the variations in the total PLA_2_ activity in the pitted areas of the peel (with respect to the healthy ones) were comparable to the changes in the expression levels of the *CssPLA_2_* genes. A similar observation was reported in citrus seedlings exposed to blue light/dark cycles, in which the total PLA_2_ activity showed the same rhythmicity as *CssPLA_2_α* gene expression [50]. In line with our findings, there are also the observations of Ryu and co-workers, who reported that the overexpression of the *AtsPLA_2_β* gene, which is orthologous to the *TdsPLA_2_I* gene, enhances tolerance of *A. thaliana* plants to salt stress [23].

As far as cereals, the only observation reported to date in the literature concerns up-regulation of the *OssPLA_2_α* gene observed in drought-stressed rice seedlings [24]. Thus, the results reported in the present study further confirm the involvement of specific *sPLA_2_* genes in the adaptation of important crops to water stress conditions. Moreover, stress-dependent induction of *TdsPLA_2_* expression was related to the increase in the total PLA_2_ activity and to the amounts of FFAs observed under the same conditions. This latter finding is consistent with previous observations that in leaves of durum wheat plants exposed to water stress, there is a decrease in membrane phospholipids that is followed by a concomitant increase in FFA content [51,52]. Linoleate and linolenate are the major fatty acids in plant membranes, and they are located preferentially at the *sn*-2 position of phospholipids. For this reason, it is feasible that their release is regulated by specific acyl hydrolases, such as the sPLA_2_s. For the slight increase in free palmitate, which is mainly esterified at the *sn*-1 position of plant phospholipids, this cannot be ascribed to the PLA_1_ activity of a generic acyl hydrolase, as the generic acyl hydrolase activity detected in durum wheat leaves was not affected by water stress. However, a role of the specific TdsPLA_2_ in liberating the palmitate cannot be excluded. In this regard, Fujikawa and co-workers have very recently characterized the recombinant enzyme encoded by the *Nt1PLA_2_* gene of *N. tabacum*, which was found to hydrolyze the ester bond at the *sn*-1 position of phospholipids, as well as at the *sn*-2 position [33]. The release of FFAs under stress conditions is consistent with their role as second messengers in the lipid signaling in plant response to adverse environmental stimuli. In particular, free linoleate and linolenate can be metabolized via the octadecanoic pathway to oxylipins, such as jasmonic acid and its methyl ester, which are essential components in the signaling pathway involved not only in plant response to pathogens and wounding, but also to adverse environmental conditions [53]. In particular, there is evidence that in barley, a species that is phylogenetically closely related to wheat, there is an increase in jasmonic acid levels in plants exposed to osmotic and salt stress [54,55].

## 4. Experimental Section

### 4.1. Plant Material and Growing Conditions

Seeds from durum wheat cultivar Ofanto were grown in a growth chamber. This genotype was chosen as it is a modern semi-dwarf cultivar well adapted to the rainfed conditions typical of the areas of the Mediterranean basin where it is widely cultivated [52]. After vernalization at 4 °C for 1 week, five seeds per pot were sown in 2.5 L plastic pots filled with soil, sand and peat (3:1:1). Twenty grams of ammonium nitrate fertilizer were applied to each pot at sowing, and a mix of mineral superphosphate (1.2 g/pot), ammonium nitrate (2.0 g/pot) and potassium sulfate (0.1 g/pot) was applied at tillering. Growth conditions varied from 10 °C day/7 °C night, 60% relative humidity, 14 h light:10 h darkness, 500 mmol m^−2^·s^−1^ photon flux density at the third leaf stage, to 30 °C day/25 °C night, 35% relative humidity, 18 h light:6 h darkness, 500 mmol m^−2^·s^−1^ photon flux density at kernel physiological maturity. Under these conditions, about 4 months were needed to achieve maturity. To evaluate the plant water status, the youngest fully expanded leaves were selected at random for each treatment, and measurements of leaf water potential (ψ_I_) were carried out using a pressure chamber (PMS Instruments Co., Corvallis, OR, USA).

As far as water treatments, pots were watered to exceed field capacity (*i.e.*, the maximum amount of water that a soil can hold) and left to drain until a constant weight was reached. In order to calculate the soil water content (SWC) at field capacity, the weight of soil of three extra pots at both water-saturated and completely dried state was measured, and the mean value obtained for the SWC at field capacity was used to calculate the amount of water to be applied to each pot. The SWC was controlled by weighing each pot every day and then, on the basis of the weight of the soil and of the empty pot determined before sowing, the amount of water needed to maintain SWC close to field capacity (33%) was added until plants reached the developmental stages chosen to impose the drought treatment: stem elongation and kernel watery ripening. At these two stages, the control plants continued to be watered so as to maintain 33% SWC, while the watering of plants assigned to the drought treatment was stopped in order to allow the soil to dry up to reach 18% SWC. The stress condition was maintained for several days by adding the amount of water needed to maintain 18% SWC until the ψ_I_ of the water-stressed plants reached −3.05 ± 0.215 MPa; control plants at the same time showed a ψ_I_ of −0.95 ± 0.096 MPa. To allow recovery of the water status, water-stressed plants were re-watered by adding the amount of water needed to restore the 33% SWC and reach a ψ_I_ of 1.05 ± 0.112 MPa, comparable to that measured in the control plants at the same time: 1.00 ± 0.095 MPa. A completely randomized design was adopted, with three replications for each treatment, as well-watered and water-stressed. For each treatment, tissue from the youngest fully expanded leaves of three randomly selected plants for replication was used for gene expression, enzyme activity, and FFA content determinations. In addition, gene expression analysis was carried out in tissues from roots, culms, glumes, seeds and awns.

### 4.2. Isolation and Sequence Analysis of the Full-Length sPLA_2_ Transcripts

A BLAST search was carried in the TIGR wheat and barley EST database and in the GenBank database, using as query the full-length transcript sequences deduced from the four genes encoding putative sPLA_2_s in *Oryza sativa* deposited in the Rice Annotation Project Database [56]: Os02g0831700 (*OssPLA_2_I*) Os03g0261100 (*OssPLA_2_II*), Os03g0708000 (*OssPLA_2_III*) and Os11g0546600 (*OssPLA_2_IV*).

The BLAST search allowed the identification in wheat of two full-length expressed sequences, CV769583 and TC381660, showing, respectively, the highest identity with *OssPLA_2_II* and *OssPLA_2_III*, and in barley of two full-length expressed sequences TC224841 and AK358216.1, showing, respectively, the highest identity with *OssPLA_2_I* and *OssPLA_2_IV*.

Total RNA was isolated from the youngest fully expanded leaves at the tillering stage using the Trizol reagent (Invitrogen), following the manufacturer instructions. The single-stranded cDNA was synthesized using 200 E.U. SuperScript™ II RNase H-reverse transcriptase (Invitrogen) and a poly(T) primer, on 1 μg total RNA, following the manufacturer’s instructions. The first strand cDNA was used as template for the amplification of the full-length sPLA_2_ transcripts using the primer pairs reported in Table 1 and high-fidelity “Phusion” Taq DNA polymerase (Finnzymes). The PCR conditions were as follows: preheating at 98 °C for 30 s, then 35 cycles of denaturation at 98 °C for 10 s, annealing at 58 °C to 62 °C for 30 s and extension at 72 °C for 30 s, followed by final extension at 72 °C for 5 min. All the PCR products were visualized on agarose gels, cloned and sequenced on both strands to confirm their identity.

The deduced protein sequences were subjected to bioinformatic analysis. Alignments were carried out using the Vector NTI Suite software (version 9.0; Invitrogen). The physico-chemical parameters of the amino acid sequences were estimated using the ProtParam tool, which is available at the ExPASy molecular biology server [57]. The conserved domains were determined using the NCBI Conserved Domain Database [27]. The putative localization was predicted by the application of the predictive tools iPSORT [58], TargetP [59] and Predotar [60].

### 4.3. Semi-Quantitative RT-PCR Analysis

Total RNA was isolated from different plant tissues using Trizol reagent (Invitrogen), following the manufacturer’s instructions. To avoid starch contamination, the seeds were ground under liquid nitrogen and the powder obtained was treated with 50 mM Tris-HCl buffer, pH 9.0, 200 mM NaCl, 1% sarcosil, 20 mM ethylenediaminetetraacetic acid, and 5 mM DTT, and subjected to phenol-chloroform extraction. The purified samples were then used for Trizol extraction. A DNase treatment step was performed at the end of the extraction to ensure the removal of genomic DNA from the total RNA extracted.

First strand cDNA was used as a template for the amplification of fragments corresponding to the *TdsPLA_2_*-expressed sequences. Normalization of the Reverse-Transcription-PCR (RT-PCR) reactions was performed by amplifying the wheat TC264064, which was 99% identical to the amino acid level of the rice *actin1* gene. A dehydrin gene, *cor410,* was amplified to monitor correct drought stress imposition.

Amplifications of fragments were performed using the specific primer pairs reported in Table 1 and Go-Taq DNA polymerase (Promega), under the following amplification conditions: preheating at 94 °C for 5 min, then 30 cycles of denaturation at 94 °C for 1 min, annealing at 58 to 67 °C for 30 s, and extension at 72 °C for 1 min, followed by a final extension of 5 min.

### 4.4. Assay of PLA_2_ Activity in the Crude Leaf Extract

PLA_2_ activity was assayed on extracts obtained by homogenization of the leaf tissue under liquid nitrogen. One gram of the powder obtained was suspended in 5 mL of cold-grinding 50 mM Na phosphate buffer, pH 7.0, and the suspension was centrifuged (twice) at 35,000 × *g* at 4 °C for 15 min. The supernatant was stored in an ice-water bath and used daily. Total protein content of the crude leaf extract was determined by the Lowry’s method as modified by Harris [61], using bovine serum albumin as the standard.

Lipid solutions (50 mM) were prepared by dissolving the appropriate amount of PC_LIN_, PC, PE, PI, PS, PG, MGDG, DGDG and TAG in ethanol containing 15% (*v*/*v*) Tween 20. PACOCF_3_ and BEL were dissolved in dimethyl sulfoxide. All of the solutions were stored in an ice-water bath and used daily.

PLA_2_ activity was evaluated using a continuous spectrophotometric method based on the PLA_2_/LOX coupled reactions, as reported by Trono *et al.*[32]. Unless otherwise specified, the assay was carried out in 2 mL of 50 mM Na borate buffer (pH 9.0) containing 4 E.U. LOX, 1.5 mM PC_LIN_ and 2 mM CaCl_2_ at 25 °C. The reaction was started with the addition of 0.1 mg crude leaf extract, and the generation of free linoleate due to PLA_2_ hydrolysis was monitored following the absorbance increase at 234 nm caused by its conversion into conjugated diene hydroperoxide catalyzed by the coupled LOX reaction (ɛ_234_ = 31 mM^−1^·cm^−1^, [62]). Control was always performed to verify that the overall rate of the reaction was not limited by LOX activity. Specific activities were expressed as E.U. per gram of fresh weight of the leaf tissue and were reported as mean value ± SD of three independent experiments.

In the experiment of the pH profile (Figure 4A) the buffers were as follows: 50 mM Na acetate, pH 4.0 and 5.0; 50 mM Na phosphate, pH 6.0–8.0; 50 mM Na borate, pH 9.0–11.5. In the experiment reported in Figure 4B, a substrate concentration of 500 μM was used, as at higher concentrations TAG caused turbidity in the reaction mixture and did not allow an accurate determination of the reaction rate.

### 4.5. Determination of FFA Content in Durum Wheat Leaves

Total lipids were extracted from 40 mg dried leaf tissue added with 1.5 mL diethyl ether:hexane (1:1, *v*/*v*), to which a known amount of heptadecanoic acid (17:0) was previously added as the internal standard. The resulting mixture was vigorously shaken and subsequently centrifuged at 1700× *g* for 10 min. The extraction was repeated three times and the organic phases were pooled in a 5 mL calibrated flask, and taken to volume with diethyl ether–hexane 1:1.

Separation of FFAs from bound fatty acids present in the lipidic extract was achieved by solid-phase extraction (SPE) procedure according to Gambacorta *et al.*[63], with some modifications. STRATA NH2 cartridges pre-packed with 500 mg of amminopropyl–silica gel (Phenomenex, Torrance, CA, USA) were used. FFAs were recovered by elution with 5 mL of diethyl ether–formic acid (2%) solution, dried under vacuum and resuspended in 200 μL diethyl ether.

Quantitative analysis of linoleate, linolenate and palmitate in the SPE fraction was carried out using gas chromatography coupled to mass spectrometry (GC-MS). A 6890N series gas chromatograph (Agilent Technologies) equipped with a ZB-FFAP capillary column (30 m × 0.32 mm i.d.; 50 μm film thickness; Restek, Bellerofonte, PA, USA) and an Agilent 5973 mass selective detector was used. The identification of FFAs was achieved by comparing mass spectra with those of the data system library (NIST 98, *p* > 95%) and retention indices with standards. Calibration graphs were prepared by the GC-MS analysis of diethyl ether solutions containing known amounts of the standards covering the range for the FFAs assayed. The linear correlation coefficient (*r*^2^) of 0.996 suggests that the method effectively addressed the quantification of FFAs. Recovery of the SPE process was determined by the analysis of the solution containing known amounts of the standards before and after the SPE extraction. The percentage recovery was >99% for all the compounds investigated.

## 5. Conclusions

Overall, our data indicate the existence in durum wheat of four genes encoding putative sPLA_2_ isoforms. Each *TdsPLA_2_* gene is differently expressed in the different tissues, and shows differently modulated expression during leaf senescence and under drought stress. This suggests a different role for each of these sPLA_2_ isoforms in both physiological metabolism and stress-induced signaling of durum wheat plants. In light of this, further studies aimed at characterizing the biochemical features and clarifying the role of each of these isoforms are needed. The characterization of purified and/or recombinant TdsPLA_2_s, together with the use of mutants lacking or overexpressing the *TdsPLA_2_* genes, will be helpful to distinguish the roles of this class of enzymes under stress and also under normal conditions.

## Figures and Tables

**Table 1:** Primer pairs used to amplify *TdsPLA_2_*, *actin1* and *cor410* transcripts, annealing temperature and PCR product sizes.

Amplicon	Forward primer (5′→3′)	Reverse primer (5′→3′)	Annealing temperature (°C)	Product size (bp)
**Full-length**				
*TdsPLA_2_I*	ATGGCGATGGCGATGGCGATG	CTACAGTTCTAACTTCTGGCTGCCC	60	426
*TdsPLA_2_II*	ATGAGATCGGTGCTCGGTC	CTACTGCCCGATGTCGCG	58	474
*TdsPLA_2_III*	ATGGCGCATGGCAGAGGC	CTAGGGCTTGTGCAGGACCCG	60	489
*TdsPLA_2_IV*	ATGCACACCGGCCGCCTCCTCCC	CTACTTCGCCGGGGCCGGCGCC	62	513
**Fragment**				
*TdsPLA_2_I*	GTCCTCCTCCTGGCCGGGGGC	CAGGCATCGAGGTCGTCGCAGGG	67	170
*TdsPLA_2_II*	TGCTTCTCCTCTCGCTGGTGACG	TCGCCGGGGCAGCCGCTGTAG	62	184
*TdsPLA_2_III*	ACGTCGGCCTCCAGACCCTCG	TCCAGCAGGCCCTCGTTGCAC	63	253
*TdsPLA_2_IV*	GCGGTACGGCAAGTACTGCGGCG	CCACGCAGTCCAGCAGGCTCTGG	66	157
*actin1*	CTTCGGACCCAAGAAAGAAAGCC	CACCGCCCGTATTTCTCTAGTAGCC	62	280
*cor410*	AGAAGAAGGAGGAGGAGGACAAGAAGAAGG	AGAAGCCTTTCTTCTCCTCCTCGGG	58	432
